# Development and application of a baculovirus-expressed capsid protein-based indirect ELISA for detection of porcine circovirus 3 IgG antibodies

**DOI:** 10.1186/s12917-019-1810-3

**Published:** 2019-03-06

**Authors:** Sujiao Zhang, Dongliang Wang, Yifan Jiang, Zhoumian Li, Yawen Zou, Meng Li, Haoyang Yu, Kun Huang, Yi Yang, Naidong Wang

**Affiliations:** Hunan Provincial Key Laboratory of Protein Engineering in Animal Vaccines, Laboratory of Functional Proteomics, Research Center of Reverse Vaccinology, College of Veterinary Medicine, Hunan Agricultural University, Changsha, 410128 China

**Keywords:** Porcine circovirus type 3, Diagnostic, Pig, ELISA

## Abstract

**Background:**

Porcine circovirus type 3 (PCV3), recently widely isolated from pigs with various clinical conditions, is likely globally epidemic. However, development of serological diagnosis for PCV3 in pigs is ongoing. Our objectives were to: 1) establish an indirect ELISA, using PCV3 capsid protein (Cap) prepared by Baculovirus Expression Vector System (BEVS) as a high-quality coating antigen for detection of PCV3-associated antibodies in serum samples; and 2) use this ELISA to conduct a serological survey for PCV3 in various regions of Hunan province, China.

**Results:**

The PCV3 positive rate to the ELISA assay (total of 190 serum samples) was higher in sows with reproductive failure compared to healthy sows (34/85, 40.0% versus 30/105, 28.6%), with similar results using qPCR assays. Further, in an additional 1038 serum samples collected from January 2016 to May 2018 in various regions of Hunan province and tested with this established ELISA, 20 to 84% were positive for PCV3 (according to region of sera collection), with high PCV3 seroprevalence (> 50%) in herds in Changde, Hengyang and Yueyang. Moreover, among serum samples from herds in Shaoyang and Changde, PCV3 seroprevalence was higher in sows than in other classes of pigs (i.e., suckling piglets, nursery pigs, gilts, growing-finishing pigs and boars).

**Conclusions:**

We developed a full-length PCV3 Cap-based ELISA using a eukaryotic expression system with excellent potential to elucidate PCV3 epidemiology. Based on this assay, PCV3 has been circulating in Hunan province. PCV3 prevalence was lower in healthy sows than in those with reproductive failure. Further studies are warranted to identify the PCV3 responsible for high seroprevalence in sows and determine pathogenesis of PCV3 in sows with reproductive failure.

**Electronic supplementary material:**

The online version of this article (10.1186/s12917-019-1810-3) contains supplementary material, which is available to authorized users.

## Background

Porcine circovirus (PCV) type 1 (PCV1) is a cell culture-derived, non-pathogenic virus, whereas PCV type 2 (PCV2) causes PCV2-associated diseases (PCVAD) for swine, a globally important pathogen causing substantial losses in the swine industry [1]. Recently, a novel porcine circovirus type 3 (PCV3) was isolated from pigs on an American farm where sows had chronic reproductive problems and clinical signs consistent with porcine dermatitis and nephropathy syndrome (PDNS) [2]. Subsequently, PCV3 was also detected from pigs, with various clinical conditions, from many countries (including China, Poland, Brazil, South Korea, Denmark, Italy and Spain) [3–9], suggesting the virus may have spread worldwide. Although PCV3 was first identified in 2015 [2], based on retrospective studies, cases of PCV3 infection were occurring as early as 1996 in pigs in China [10]. Based on recent reports using PCR, co-infections of PCV2 and PCV3 are common in pigs [5, 11–14]. Furthermore, there are indications that co-infections of PCV3 with other pathogens (e.g. PRRSV) may increase pathogenicity in pigs [2, 15]. In most reports, the PCV3 genome was detected and cloned from diseased pigs, which suggested PCV3 was likely associated with PDNS, reproductive failure, cardiac and multisystemic inflammation [2, 4, 5]. Additionally, there is a report on PCV3 infection in pigs without any significant clinical signs or symptoms [16, 17]. Susceptibility of wild boars to PCV3 (without clinical signs) was also reported [18, 19]. Furthermore, it was recently confirmed that PDNS-like disease was induced by experimental PCV3 infection of 4- and 8-wk-old piglets [20]. Based on PCV3 detection in semen, sow colostrum and tissues from stillborn piglets, PCV3 infection in aborted fetuses was due to vertical transmission [2, 21–23], implicating this route in spread of PCV3 [2, 24]. That PCV3 was present in clinical serum samples in dogs with various clinical signs [25], indicates potential risks of cross-species transmission, with implications for public health.

The PCV3 genome comprises 2000 nucleotides (nt), containing three major, inversely arranged open reading frames (ORFs). ORF1 and 2 are separated by a conserved stem-loop structure consisting of 9-nt (TAGTATTAC), identical to that of PCV1 [2]. PCV3 capsid protein (Cap, 214 amino acids, aa), encoded by ORF2 is 19~20 aa shorter than PCV2 Cap. Notably, both Caps share only 37% identity of aa residues, although they have a very similar jelly-roll fold [5].

To date, PCV3 Cap without a nuclear localization signal (NLS) sequence at the N-terminal end was prepared from bacteria and used in an ELISA to detect the presence of PCV3 antibodies in swine sera [2, 26]. Although this confirmed that the Cap was a suitable target antigen for detection of PCV3-specific antibody, it was noteworthy that viral antigens prepared from a baculovirus/insect cell system have many advantages over other systems, including high yields, various post-translational modifications and ability to mimic antigenic properties of their viral counterparts.

The N terminus of NLS sequence of PCV3 Cap is rich in arginine residues encoded by low-usage codons in *E. coli*. In our previous study, peptides in the PCV3 Cap potentially recognized by B and T cells were identified using the Immune Epitope Database Analysis Resource (IEDB) (http://www.iedb.org) [27]. We determined that a B-cell epitope (aa 10–18) and T-cell epitope (aa 29–38) were located in the NLS sequence of PCV3 Cap (Additional file 1: Table S1). Moreover, the B-cell epitope (aa 10–18) was conserved in the Cap NLS of all PCV3 reference strains (Additional file 1: Figure S1). Therefore, both the NLS-truncated and the full-length Caps may have different reactions with PCV3 antibody in pig serum. Interestingly, a peptide (aa 17–50) located within the NLS of PCV2 Cap was speculated to be an important epitope in a previous report [28]. Later, a core epitope (^26^RPWLVHPRHRY^36^) located in the NLS region of PCV2 Cap was also identified by monoclonal antibodies [29]. Therefore, peptides located in the NLS of PCV3 Cap should be considered critical targets in developing an ELISA for serodiagnosis and vaccine design. We expect that the PCV3 capsid protein (Cap) prepared by Baculovirus Expression Vector System (BEVS) as a coating antigen will be useful for development of a reliable ELISA for PCV3 antibody detection and going forward, evaluating efficacy of PCV3 vaccine in clinical samples.

Our objectives were to: 1) prepare a core antigen containing full-length PCV3 Cap via Baculovirus Expression Vector System; 2) use this Cap as a coating antigen to establish an indirect ELISA for detection of PCV3-specific antibody in swine sera; and 3) use this ELISA to conduct a serological survey for PCV3 prevalence on farms in various regions of Hunan province.

## Results

### Preparation of PCV3 cap

**Fig. 1:**
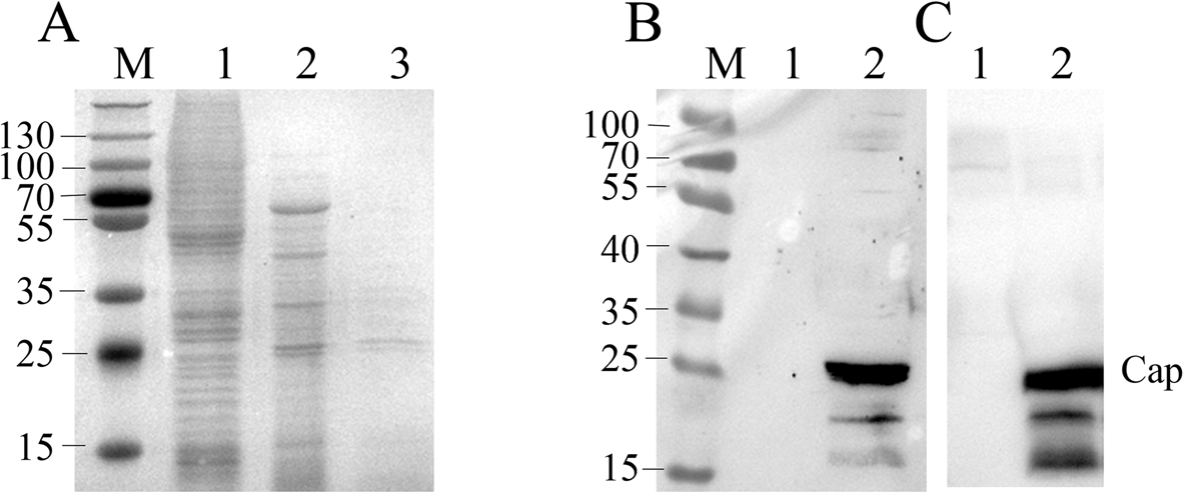
Expression and purification of PCV3 Cap with a Baculovirus expression system. PCV3 Cap was expressed in Sf9 cells and purified with a Ni-NTA resin, separated on a 12% SDS-PAGE gel and stained with Coomassie blue (**a**), and identified using western blot with swine PCV3-specifc positive serum (**b**) and anti-His tag antibody (**c**). M, protein marker in KDa. Lane 1: Total extract in non-infected SF9 cells. Lane 2: Total extract in Sf9 cells expressed PCV3 Cap; Lane 3 Ni-NTA affinity-purified Cap protein

After recombinant baculovirus containing full-length of PCV3 *cap* gene was successfully constructed and amplified in a Bac-to-Bac baculovirus expression system, expression of PCV3 Cap in Sf9 cells was successfully identified by SDS-PAGE and western blot using swine PCV3 positive serum and anti-His tag antibody, respectively. A clear band revealed that PCV3 Cap with an apparent molecular weight of ~ 26 KDa was present in cell lysates from Sf9 cells infected by a recombinant baculovirus (Lane 2 in Fig. 1B and C), but not in the Control (Lane 1 in Fig. 1B and C). Subsequently, PCV3 Cap (40 μg/mL) was successfully purified from the cell lysates via Ni-NTA affinity chromatography (Lane 3 in Fig. 1A).

### Establishment of ELISA using purified PCV3 cap as antigen

**Fig. 2:**
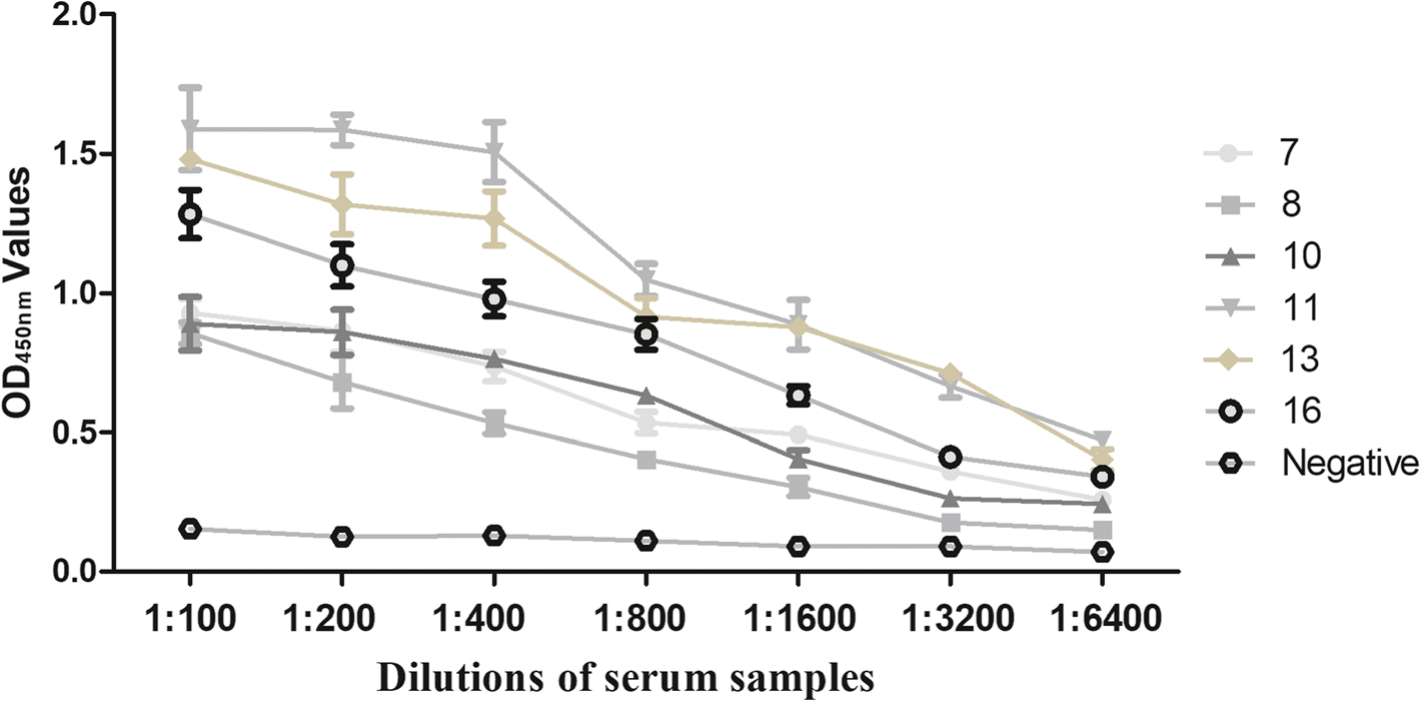
ELISA sensitivity using PCV3 Cap protein as coating antigen. Six PCV3 positive and one negative serum samples were serially titrated and tested for reactivity in the PCV3 Cap based ELISA.

A checkerboard titration was performed to determine the optimal Cap concentration for plate coating and serum dilution in the ELISA. Based on our results (Table 1), 0.5 μg/mL of Cap for plate coating, along with a serum dilution of 1:100 was an optimal combination and chosen for subsequent ELISA assays. Furthermore, the optimal dilution of the secondary antibody was 1:7000 (Additional file 1: Table S3). The OD450 nm values decreased with successive dilutions of six positive serum samples, indicating that our PCV3 Cap ELISA had high sensitivity (Fig. 2). All five non-PCV3 serum samples used to determine specificity were negative for PCV3 Cap ELISA (Additional file 1: Table S4).

**Table 1:** Optimal dilutions of coating antigen and serum sample antibodies for ELISA

Serum dilution	Concentration of coating antigen (X ± SD, μg/mL)				
	0.25	0.5	1	2	3
50× (+)	1.043 ± 0.021	1.076 ± 0.028	1.306 ± 0.061	1.252 ± 0.068	1.288 ± 0.060
50× (−)	0.189 ± 0.006	0.213 ± 0.002	0.293 ± 0.004	0.283 ± 0.012	0.352 ± 0.087
P/N	5.519	5.051	4.457	4.424	3.659
100× (+)	0.812 ± 0.043	1.144 ± 0.032	1.223 ± 0.159	1.213 ± 0.086	1.086 ± 0.090
100× (−)	0.186 ± 0.005	0.197 ± 0.023	0.269 ± 0.024	0.286 ± 0.050	0.323 ± 0.098
P/N	4.366	5.807	4.584	4.241	3.362
150× (+)	0.800 ± 0.069	1.044 ± 0.076	0.922 ± 0.006	0.876 ± 0.048	0.986 ± 0.030
150× (−)	0.173 ± 0.016	0.185 ± 0.033	0.235 ± 0.001	0.256 ± 0.023	0.313 ± 0.008
P/N	4.624	5.643	3.923	3.422	3.150
200× (+)	0.742 ± 0.064	1.019 ± 0.146	0.776 ± 0.130	1.143 ± 0.073	1.125 ± 0.009
200× (−)	0.163 ± 0.010	0.241 ± 0.051	0.225 ± 0.001	0.273 ± 0.002	0.276 ± 0.009
P/N	4.552	4.228	3.449	4.187	4.076

**Fig. 3:**
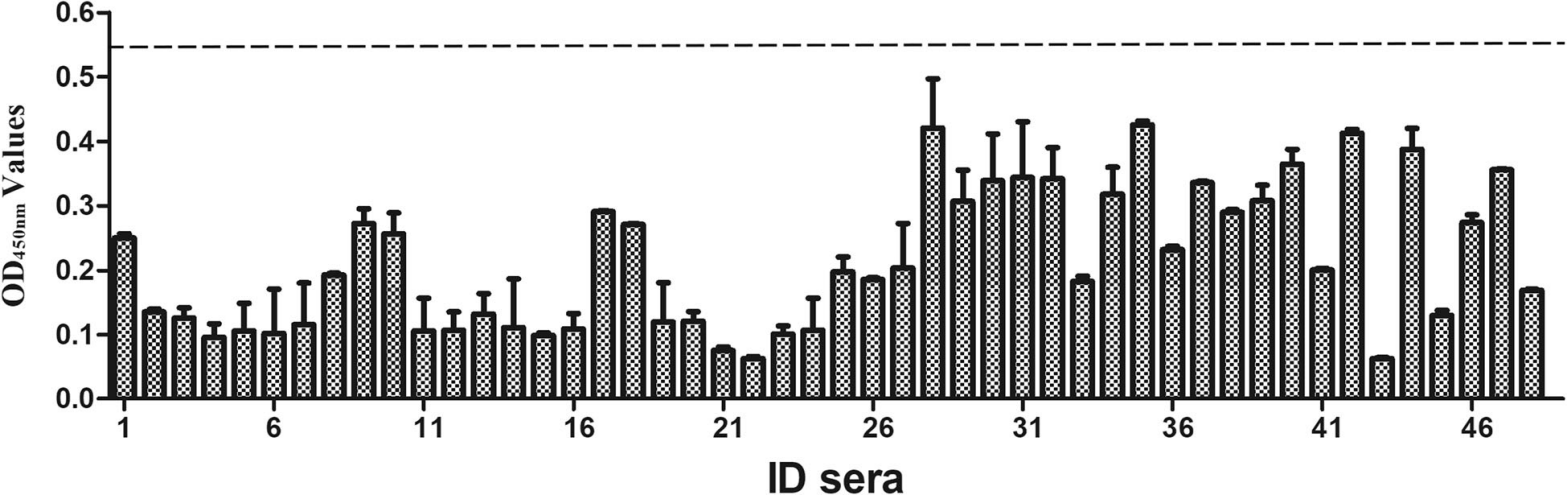
Determination of cut-off value for PCV3 Cap ELISA. Cut-off values were evaluated by testing 48 negative control sera. Each value represented the mean absorbance at 450 nm obtained from three replicates of each serum sample tested. The cut-off value was defined as mean OD value of all tested negative control sera, plus three standard deviations. The cut-off value (0.541) was represented by dashed lines. Error bars indicated mean and SD

The cutoff value was determined by the sum of the mean OD450 nm value (X) and three standard deviation (SD) of negative sera. All 48 qPCR negative serum samples were further verified by IFA to confirm that they were negative for anti-PCV3 antibodies (Additional file 1: Figure S2). Therefore, these sera samples were used to determine the cut-off value (Fig. 3); they had an average absorbance of 0.214, with standard deviation 0.109. The ELISA threshold was 0.214 + 3 × 0.109 = 0.541.

### Comparisons of PCV3 antibody and viremia levels

**Fig. 4:**
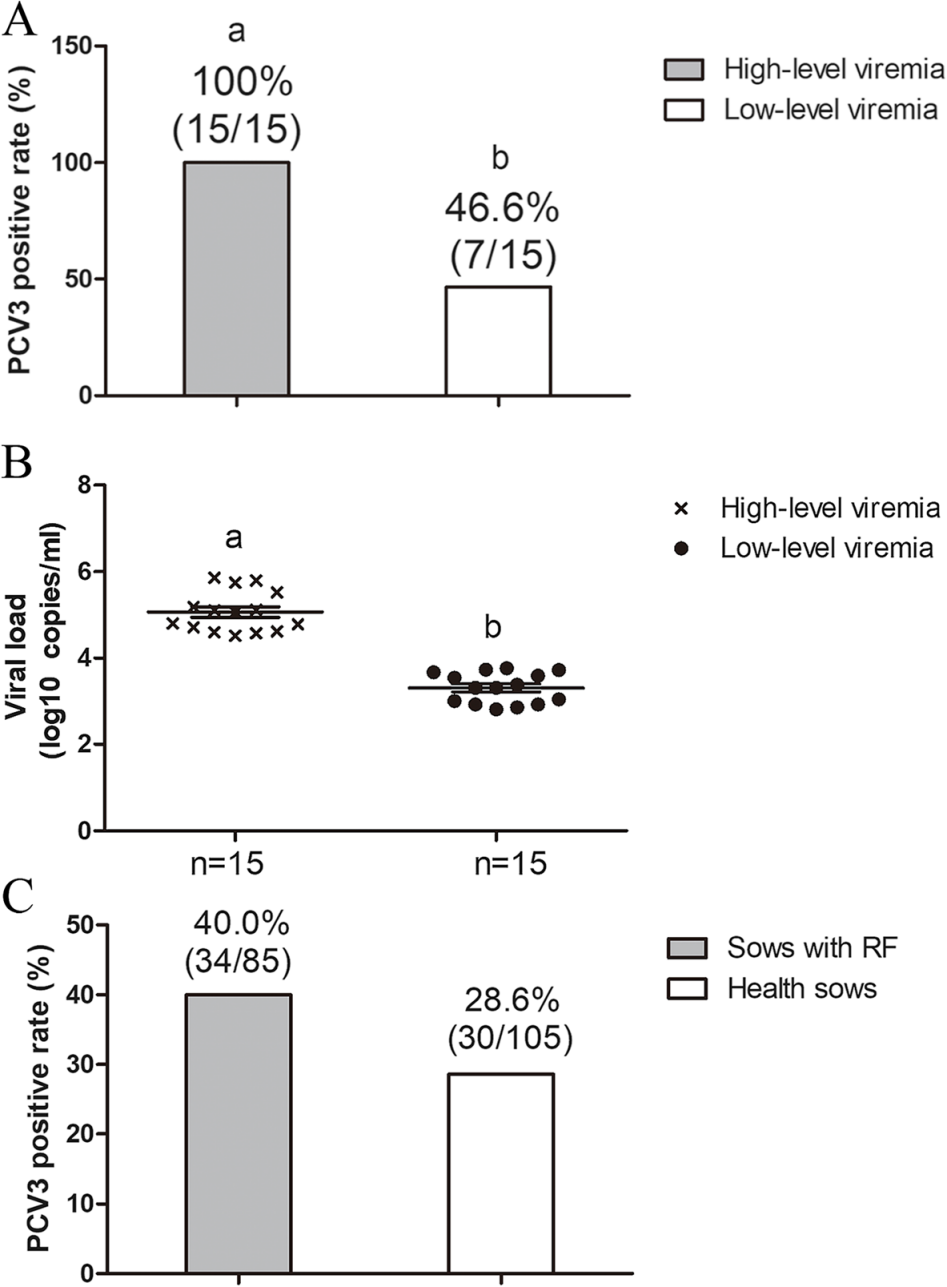
A PCV3 Cap ELISA was used to compare PCV3 antibody with viremia level of serum samples and determine PCV3 positive rate of healthy sows versus sows with RF (reproductive failure). (A and B) Comparisons of PCV3 antibody and viremia levels; (C) PCV3 positive rate of healthy sows and sows with RF

The PCV3 qPCR-positive serum samples (*n* = 30, as described [27]) were divided into two groups (high- and low-level viremia) based on level of viremia. Copy numbers of virus genomic DNA in serum samples of high- and low-level viremia groups ranged from 3.30 × 10^4^ to 7.34 × 10^5^ and from 6.49 × 10^2^ to 5.84 × 10^3^ copies/mL, respectively (Table 2). The PCV3 antibody-positive rate in the serum samples with high-level viremia was significantly higher than that with low-level viremia. Overall, the PCV3 prevalence based on detection by qPCR (45.9%, 39/85) as described [27] and ELISA (40%, 34/85) in sows with reproductive failure were significantly higher than that in healthy sows [21.9 (23/105), [27] and 28.6% (30/105), respectively; Fig. 4] among the 190 sow serum samples. These results confirmed the efficiency of our PCV3 ELISA.

**Table 2:** The PCV3 viral load of positive serum samples

Group	Sample	PCV3 viral load (copies/mL)
High-level viremia	9	1.15 × 10^5^
	74	1.53 × 10^5^
	2	1.3 × 10^5^
	13	1.27 × 10^5^
	15	6.08 × 10^4^
	6	4.14 × 10^4^
	72	6.32 × 10^4^
	18	5.13 × 10^4^
	1–1	3.30 × 10^4^
	78	3.78 × 10^4^
	42–1	3.93 × 10^4^
	24	3.36 × 10^5^
	99	6.24 × 10^5^
	231	7.34 × 10^5^
	657	5.65 × 10^5^
Low-level viremia	113	2.05 × 10^3^
	001	6.49 × 10^2^
	590–124	8.44 × 10^2^
	2–1	7.17 × 10^2^
	32	2.42 × 10^3^
	79	1.1 × 10^3^
	25	8.39 × 10^2^
	55	1.01 × 10^3^
	8	3.88 × 10^3^
	13	4.66 × 10^3^
	113	2.05 × 10^3^
	92–1	5.38 × 10^3^
	67	3.51 × 10^3^
	23	5.84 × 10^3^
	43	5.25 × 10^3^

### Serological evidence for PCV3 infection on farms from various regions in Hunan province

**Fig. 5:**
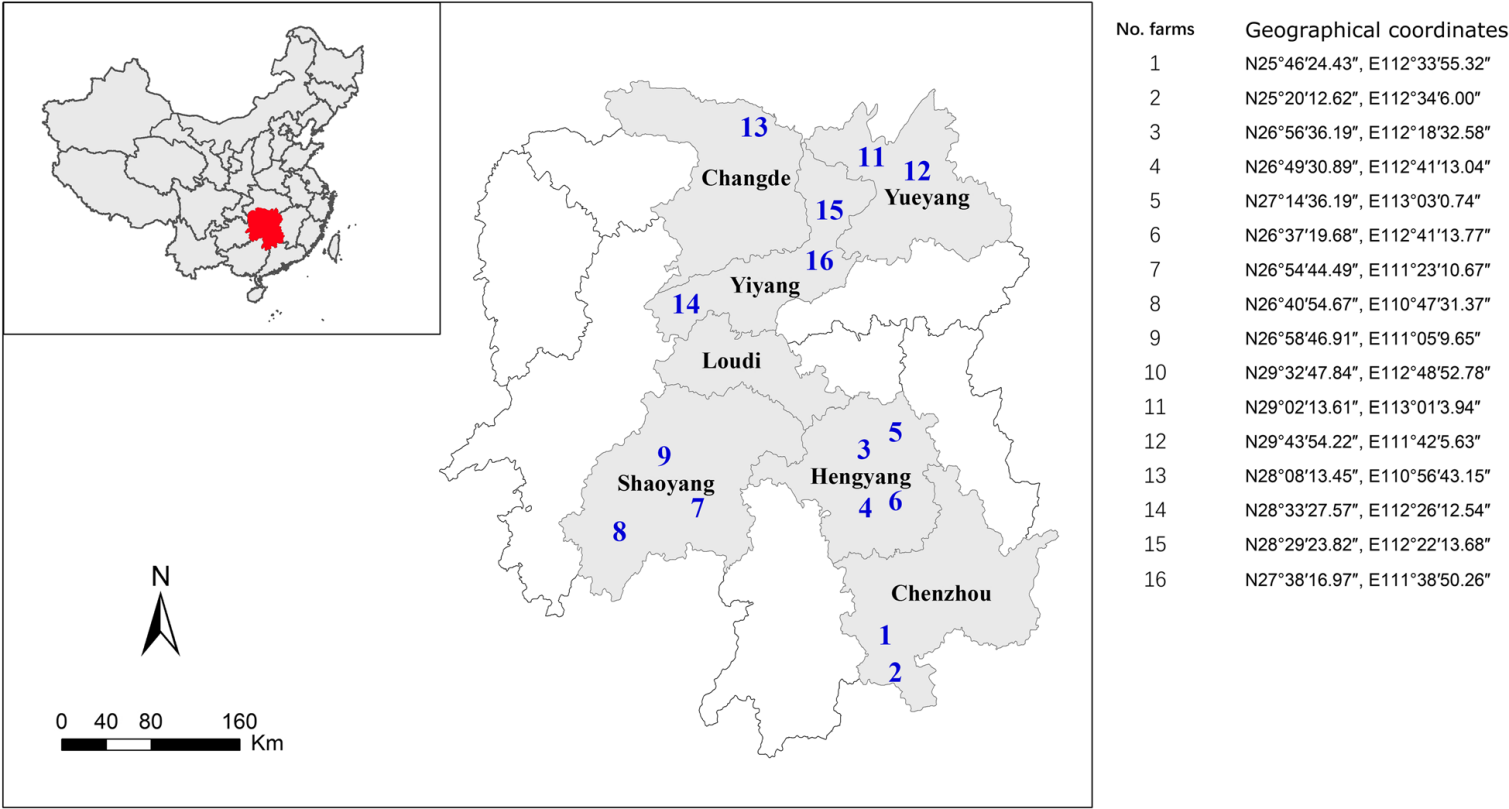
Map (produced in-house), with location of Hunan province in China highlighted in red and locations of various regions (grey indicates location with PCV3 positive samples) in Hunan province where porcine serum samples were collected from 2016 to 2018

**Fig. 6:**
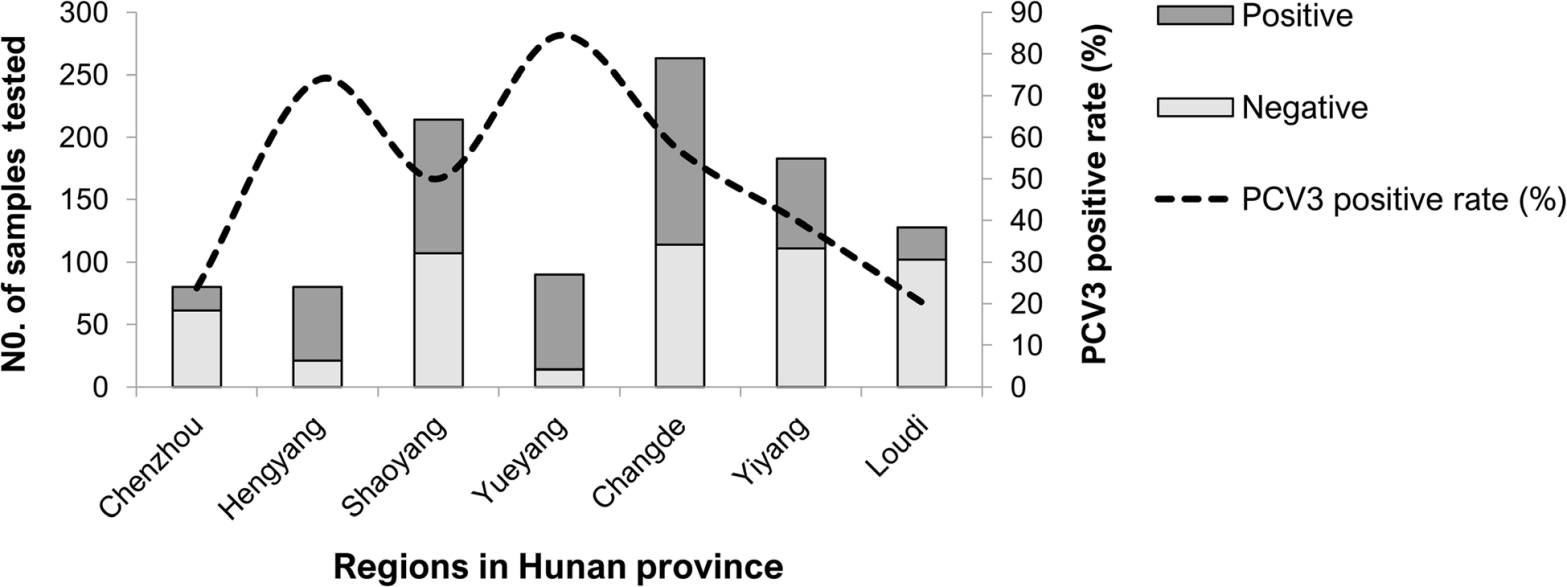
PCV3 positive rates on farms in Hunan province

Based on ELISA results of 1038 serum samples, PCV3 has been circulating in Hunan province (Fig. 5). Seroprevalence of PCV3 on farms of Hunan province was 49% (95% CI:45.9–52) and widespread from 20% (95% CI:13.2–27.4) to 84% (95% CI:76.8–92.1). Areas of Loudi, Chenzhou, Yiyang and Shaoyang had a seroprevalence < 50%, whereas PCV3 seroprevalence was higher (> 50%) in herds in Changde, Hengyang and Yueyang (Fig. 6, Table 3). Furthermore, PCV3 seroprevalence was higher in sows than in other classes of pigs [96% (68/71) and 65% (60/93), respectively] in herds from Shaoyang and Changde (Additional file 1: Table S5).

**Table 3:** Origin of serum samples used in this study

Regions	No. farms	No. samples tested	No. positive	Positive rate (%) (95%CI)
Chenzhou	2	80	19	24 (14.2–33.3)
Hengyang	4	80	59	74 (63.9–83.6)
Shaoyang	3	214	107	50 (43.2–56.8)
Yueyang	2	90	76	84 (76.8–92.1)
Changde	1	263	149	57 (50.6–62.7)
Yiyang	3	183	72	39 (32.2–46.5)
Loudi	1	128	26	20 (13.2–27.4)
Total	16	1038	508	49 (45.9–52)

## Discussion

By optimizing the full-length *cap* gene of PCV3 in our study, the PCV3 Cap was well expressed in BEVS. Further, this Cap was rapidly purified via one-step NTA-Ni affinity chromatography (Fig. 1). Importantly, Cap was exploited as a coating antigen responsible for detecting PCV3-specific antibodies present in serum samples (Figs. 2, 4, 6). Previous studies also used a truncated PCV3 Cap harvested from bacteria expression system as a coating antigen to detect PCV3 antibodies in sera [26]. Of note, the NLS, an arginine-rich domain at the NH_2_-terminal end of the Cap, was deleted in the truncated Cap. The difference of antigenicity between full-length and NLS-truncated PCV3 Caps remains to be determined, as epitope mapping of PCV3 Cap has yet to be reported. Epitope prediction strongly indicated presence of a potential linear epitope in the NLS of PCV3 Cap (Additional file 1: Table S1 and Figure S1). In contrast, the NLS of PCV2 Cap contains an important epitope, whereas removal of the NLS from the Cap dramatically increased expression level of this protein in bacteria [28, 29]. We also tried to express full-length PCV3 Cap in bacteria using codon-optimized *cap* genes, but no protein of interest was detected in cell lysates (data not shown). Based on these results, we inferred that the arginine-rich NLS is a rate-limiting factor responsible for PCV3 Cap expression in bacteria.

So far, no PCV3 commercial vaccine has been marketed; therefore, we inferred that the presence of PCV3 Cap-specific antibody in our ELISA assays resulted from clinical infections of this pathogen. To validate the accuracy/specificity of this ELISA for serodiagnosis of PCV3, we used two quality controls (QC): 1) the coating antigen (purified PCV3 Cap, Fig. 1A) in Sf9 cell lysates was specifically recognized by anti-PCV3 sera (Fig. 1B); and 2) diagnostic results for our ELISA were quite consistent with those using qPCR for the PCV3 genome. All serum samples with a high-level viremia were diagnosed as PCV3 positive by our ELISA; however, in the sera with low-level viremia, 7 of 15 were identified as PCV3 positive by the ELISA. In previous reports, PCV3 titer or genomic copy number from clinical serum samples varied from 10^2^ to 10^4^ [2], much lower than that of PCV2 (> 10^7^) [30–32]. Low-level viremia indicates a lower virus titer in serum. In general, antibody level in serum is correlated to the amount and antigenicity of viral/antigen protein(s). Thus, the PCV3 Cap-specific antibody in sera with low-level viremia may not be detected due to the amount of antibody elicited by extremely low antigen concentrations. In addition, PCV3 antigen may be neutralized in low-level viremia pigs, so sera titers would be high. The relationship between viremia level and antibody level is probably more complex than previously considered. Therefore, in future studies, our established ELISA should be used for assessing sera from swine with low-level viremia.

Using the established ELISA assay, the PCV3 positive rates (*n* = 1038) increased from 20 to 69% in Hunan province between 2016 and 2018 (Additional file 1: Table S6), based on 1 y of sera collection. In contrast, serological survey of PCV3 in China, the positive rate of PCV3 increased from 22.35 to 51.88% between 2015 and mid-2017, indicating PCV3 was widespread and increasing in prevalence in pigs in China [26].

## Conclusions

We developed a full-length PCV3 Cap-based ELISA with excellent potential to elucidate PCV3 epidemiology. The baculovirus-expressed and affinity-purified PCV3 Cap may be exploited as a coating antigen for detecting PCV3-specific antibody present in sera. Based on this assay, PCV3 has been circulating in Hunan province. Finally, PCV3 prevalence was lower in healthy sows than in those with reproductive failure.

## Methods

### Serum samples

Hunan province is located in the middle of China and contains the middle reaches of the Yangtze River, lying between longitude 108,047/ and 114,015/E (Fig. 5). The pig population of the entire province was 59.2 and 61.16 million in 2016 and 2017, respectively, among the top three in China. From 2016 to 2018, serum samples (*n* = 1038, ~ 100–300 μL) were collected from commercial herds in seven regions of Hunan Province (Table 3, Fig. 5) by veterinarians from the Hunan Provincial Key Laboratory of Animal Vaccine & Protein Engineering. No aggressive operation was conducted against pigs for sampling purpose and no pigs were euthanized in this study. Additionally, another 190 serum samples (300 μL) were collected from sows with or without reproductive failure, among which copy numbers of PCV3 genome based on quantitative PCR (qPCR) assays were tested in our previous report [27]. Further, these 190 serum samples were used to determine prevalence of PCV3 viremia and antibodies in this study. Detailed information for these serum samples is summarized in Additional file 1: Table S2. To determine the correlation between viremia level and genome copy number, 30 PCV3 qPCR-positive samples among the 190 serum samples with distinct genome copy numbers were chosen.

### Expression and purification of PCV3 cap

A PCV3 *cap* gene (GenBank accession number. AQZ26221) was optimized and synthesized by GenScript Company (Nanjing, China). For gene optimization, OptimumGene™ algorithm was used to produce a single gene that can reach the highest possible level of expression. According to optimization analysis, the codon usage bias in *Spodoptera frugiperda* was increased by upgrading the Condon Adaptation Index (CAI) to 0.95, in terms of high gene expression level. A 6 × His-tag was fused to the NH_2_-terminal end of the Cap to aid protein purification. The recombinant full-length PCV3 *cap* fragment was amplified by PCR with forward primer (5′ CCGGAATTCATGCGCGGTTCCCACCACCACCACCATCACATGCGCCACCGCGCC 3′) and reverse primer (5′ CCGCTCGAGTTACAGCACGGACTTGTAGCGGATC 3′), then the target gene (675 bp) was subsequently cloned into an expression vector of pFastBac1 (Invitrogen, Life Technologies, Carlsbad, CA, USA) via two restriction sites (*EcoR* I and *Xho* I). A Bac-to-Bac baculovirus expression system (Invitrogen) was used for preparations of recombinant baculovirus and protein expression of the PCV3 Cap, in accordance with manufacturer’s instructions. Insect Sf9 cells were infected with recombinant baculovirus using an MOI of 5 for protein expression.

Cells infected with recombinant baculovirus carrying the PCV3 *cap* gene (RbPCV3-Cap) were harvested 4 d post infection (dpi). The PCV3 Cap protein was purified by Ni-NTA affinity, as described [33]. Proteins from infected cells were separated by 10% sodium dodecyl sulfate polyacrylamide gel electrophoresis (SDS-PAGE; Bio-Rad, Hercules, CA, USA), followed by Coomassie blue staining or Western blot analysis using anti-His tag antibody (1:5000, Abmart, China) and swine PCV3-positive swine serum (with strong reactive serum) from Hunan province of China that was tested with the new Cap ELISA, respectively. In addition, mock-infected Sf9 cells were lysed and used as a negative control.

### ELISA conditions

To establish the PCV3 Cap-based ELISA, antigen concentrations, serum and HRP-labeled second antibody were optimized, according to our previous report [33]. Briefly, purified PCV3 Cap was coated onto a 96-well ELISA microplate (Corning Inc., Corning, NY, USA) at 4 °C overnight and blocked with 5% skim milk for 3 h at 37 °C. Then, wells were washed five times with PBST (phosphate-buffered saline, PBS containing 0.05% of Tween 20). Thereafter, diluted clinical serum samples, negative, positive and blank samples (100 μL per well) were added into wells and incubated for 30 min at 37 °C and wells were washed five times with PBST. Subsequently, HRP-labeled goat anti-pig IgG (KPL Inc., Gaithersburg, MD, USA) was incubated in wells for 15 min at 37 °C, after which wells were washed five times with PBST and 50 μL of tetramethyl-benzidine (TMB, Sera Care Life Sciences, Inc., Milford, MA, USA) was incubated in the dark for 5 min at room temperature. Finally, 50 μL of 2 M H_2_SO_4_ was used to stop the chemical reaction. Absorbance (450 nm) was measured using an ELISA reader (BioTek, Winooski, VT, USA).

Optimal dilutions (Table 1) of the PCV3 Cap and test pig sera in the ELISA were determined by checker board titration. Various concentrations of PCV3 Cap (0.25, 0.5, 1, 2 and 3 μg/ml) were coated onto 96-well plates with 50 mM NaHCO_3_ (pH 9.6) coating buffer. In addition, sera were diluted in PBST at 1:50, 1:100,1:150 and 1:200 (*v*/v), and HRP-labeled secondary antibody was diluted to 1:4000, 1:5000, 1:6000 and 1:70000 in PBST. Optimal concentrations of antigens, sera and secondary antibody were determined based on OD450 nm values and P/N ratios (OD450 nm of positive serum/OD450 nm of negative serum). Optimal dilutions of Cap and serum to be used in the ELISA were chosen on the basis of the highest P/N ratio obtained from each combination, with an OD450 of positive serum closest to 1.0. Then the optimal concentration of secondary antibody producing the maximum P/N ratios was determined using optimal ELISA conditions noted above.

PCV3-free negative sera (100 μL, diluted at 1:100), originally determined by quantitative PCR (qPCR) for copy numbers of PCV3 genomic DNA in our previous reports [27], were further confirmed by immunofluorescence assays (IFAs), based upon intracellular PCV3 Cap expression as reported [34], using anti-PCV3 antibody-positive and negative pig sera. Briefly, for generation of intracellular PCV3 Cap expression, PCV3 *cap* gene (GenBank accession number AQZ26221) was cloned into the plasmid pCMV-HA (Clonetech, Mountain View, CA, USA), and plasmid DNA was transfected into Vero cells (ATCC, CCL-81) with Lipofectamine 2000 (Invitrogen, Burlington, ON, Canada). Then, all 48 negative sera that were further verified as negative for anti-PCV3 antibodies (based on IFA) were used to determine a cutoff value, according to PCV3 ELISA conditions described above.

Antibodies against porcine parvovirus (PPV), porcine pseudorabies virus (PRV), porcine reproductive and respiratory syndrome virus (PRRSV), classical swine fever virus (CSFV) and porcine circovirus 2 (PCV2) which were originally used for evaluation of PCV2 virus like particles (VLPs) ELISA in our previous report [33], were used to evaluate specificity of the new ELISA. Then, 100 μL aliquots (diluted 1:100) of six PCV3 positive selected for antibody titres ranging from average to very high (qPCR-positive) and one negative sera (absorbance of 0.162 ± 0.008, qPCR-negative) from the Changde region of Hunan Province were used to determine assay sensitivity.

### Statistical analyses

Overall comparisons of PCV3 positive rate and virus load in serum samples were analyzed using a Fisher’s Exact test and Kruskal-Wallis H test, respectively, using SPSS software. Statistical significance was set at *p* < 0.05 and Confidence Intervals determined. Results are presented as means±SD.

## Additional files

Additional file 1: **Figure S1.** Comparative sequences alignment of the PCV3 Cap. **Figure S2.** Indirect fluorescence assay (IFA) of Cap protein in Vero cells. **Table S1.** Epitopes of PCV3 NLS region. **Table S2.** Detailed information for 190 sow serum samples from seven farms in Hunan, China. **Table S3.** Optimal dilutions of secondary antibodies for ELISA. **Table S4.** OD450 value of PCV3 ELISA for other pathogens. **Table S5.** Description of swine in Shaoyang and Changde sampled for this study. **Table S6.** Annual summary of serum samples used in this study. (DOCX 790 kb)

## Abbreviations

BEVS: Baculovirus Expression Vector System
Cap: Capsid protein
CSFV: Classical swine fever virus
dpi: Days post infection
ELISA: Enzyme-linked immunosorbent assay
IEDB: Immune Epitope Database Analysis Resource
IFA: Indirect immunoinfluscent assay
NLS: Nuclear localization signal
ORFs: Open reading frames
PCV2: Porcine circovirus 2
PCV3: Porcine circovirus type 3
PCVAD: PCV2-associated diseases
PDNS: Porcine dermatitis and nephropathy syndrome
PPV: Porcine parvovirus
PRRSV: Porcine reproductive and respiratory syndrome virus
PRV: Porcine pseudorabies virus
SDS-PAGE: Sodium dodecyl sulfate polyacrylamide gel electrophoresis
TMB: Tetramethylbenzidine
