# Supplementary material for: Development and application of a baculovirus-expressed capsid protein-based indirect ELISA for detection of porcine circovirus 3 IgG antibodies
Source: BMC Vet Res. 2019 Mar 6;15:79. doi: 10.1186/s12917-019-1810-3 (PMC6404275; doi:10.1186/s12917-019-1810-3)
Supplement: Supplementary file 1 — Figure S1. Comparative sequences alignment of the PCV3 Cap. Figure S2. Indirect fluorescence assay (IFA) of Cap protein in Vero cells. Table S1. Epitopes of PCV3 NLS region. Table S2. Detailed information for 190 sow serum samples from seven farms in Hunan, China. Table S3. Optimal dilutions of secondary antibodies for ELISA. Table S4. OD450 value of PCV3 ELISA for other pathogens. Table S5. Description of swine in Shaoyang and Changde sampled for this study. Table S6. Annual summary of serum samples used in this study. (DOCX 790 kb) [file 12917_2019_1810_MOESM1_ESM.docx]

**Table S1.** Epitopes of PCV3 NLS region.

| **Position** | |  | **Residue** |
| --- | --- | --- | --- |
| *B cell epitopes* | | |  |
|  | 10-18 | | RPRPRRRRR |
| *T cell epitopes* | | |  |
|  | 29-38 | | FIRRPTAGTY (3.3)^a^ |

Note: ^a^ A low percentile rank “indicates higher binding affinity predicted” by IEDB





**Figure. S1.** Comparative sequences alignment of the PCV3 Cap.


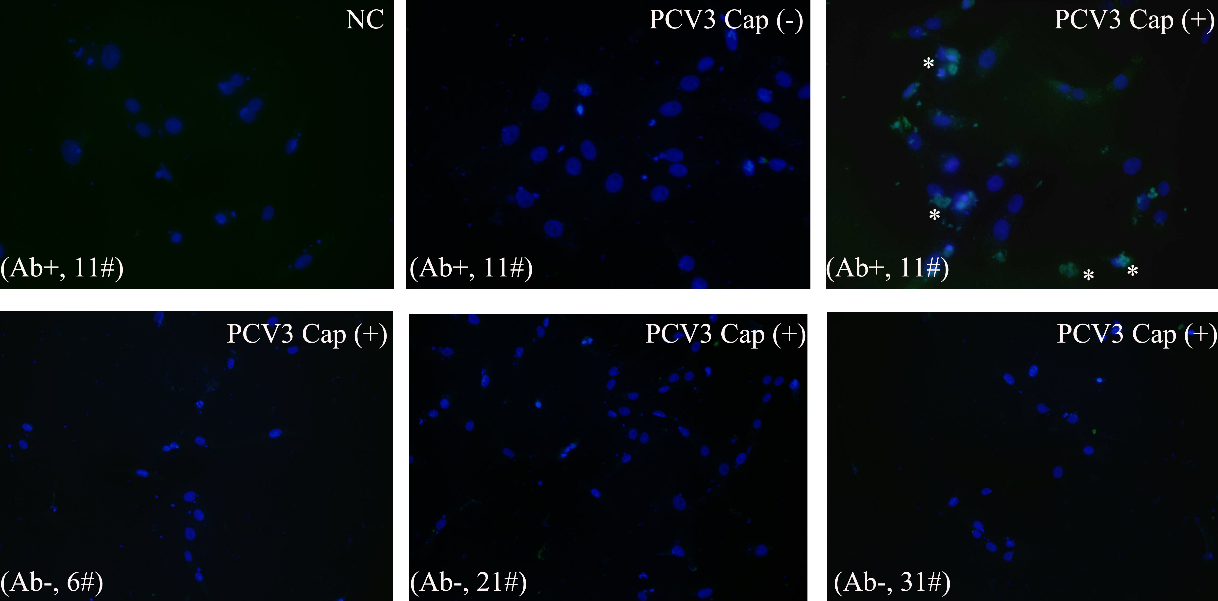


**Figure. S2.** Indirect fluorescence assay (IFA) of Cap protein in Vero cells. Recombinant construct with PCV3 Cap under CMV promoter was transfected into Vero cells. Cells were collected after 48 h and analyzed by IFA. Representative images of three independent experiments are shown. Intracellular expressed PCV3 Cap incubated with PCV3 swine negative (Ab-) and positive sera (Ab+), FITC-conjugated goat anti-pig IgG produced green fluorescence (*) in Vero cells; PCV3 Cap (-): blank plasmid transfected into Vero cells, without PCV3 Cap expression; PCV3 Cap (+): with PCV3 Cap expression. Cells were stained with DAPI (blue); NC: Vero cells.

**Table S2. Detailed information for 190 sow serum samples from seven farms in Hunan, China; these samples were originally used for PCV3 viremia level detection, based on qPCR assays [7].**

| Farm | No. sow serum samples tested | |
| --- | --- | --- |
|  | With RF | Without RF |
| A | 8 | 15 |
| B | 9 | 15 |
| C | 26 | 15 |
| D | 7 | 15 |
| E | 20 | 15 |
| F | 5 | 15 |
| G | 10 | 15 |
| Total | 85 | 105 |

RF = reproductive failure.

**Table S3** Optimal dilutions of secondary antibodies for ELISA.

| Secondary antibody dilution | Positive serum dilution (100×) |  | Negative serum dilution (100×) | P/N |
| --- | --- | --- | --- | --- |
| 1:4000 | 1.627±0.025 |  | 0.453±0.013 | 3.592 |
| 1:5000 | 1.261±0.033 |  | 0.260±0.009 | 4.850 |
| 1:6000 | 1.152±0.041 |  | 0.245±0.011 | 4.702 |
| 1:7000 | 1.109±0.029 |  | 0.212±0.008 | 5.231 |
| 1:8000 | 0.972±0.023 |  | 0.203±0.016 | 4.788 |
| 1:9000 | 0.741±0.019 |  | 0.151±0.004 | 4.907 |

**Table S4. OD_450_ value of PCV3 ELISA for other pathogens.**

| **Pathogen** | **OD_450_** |
| --- | --- |
| PPV | 0.067±0.053 |
| PRV | 0.106±0.042 |
| PRRSV | 0.098±0.036 |
| CSFV | 0.081±0.037 |
| PCV2 | 0.077±0.028 |

**Table S5. Description of swine in Shaoyang and Changde sampled for this study.**

| **Region** | **Animal class** | **No. samples tested** | **No. positive** | **Positive rate (%)** |
| --- | --- | --- | --- | --- |
| Shaoyang | Suckling piglets | 10 | 5 | 50 |
|  | Nursery pigs | 10 | 6 | 60 |
|  | Gilts | 10 | 6 | 60 |
|  | Boars | 16 | 0 | 0 |
|  | **Sows** | **71** | **68** | **96** |
| Changde | Suckling piglets | 15 | 5 | 33 |
|  | Nursery pigs | 15 | 3 | 20 |
|  | Gilts | 36 | 14 | 39 |
|  | Boars | 13 | 2 | 15 |
|  | Growing-finishing pigs | 75 | 38 | 51 |
|  | **Sows** | **93** | **60** | **65** |

**Table S6.** Annual summary of serum samples used in this study.

| **Year** | **No. samples tested** | **No. positive** | **Positive rate (%)**  **(95% CI)** |
| --- | --- | --- | --- |
| 2016 | 128 | 26 | 20（13.2-27.4） |
| 2017 | 316 | 70 | 22（17.4-26.6） |
| 2018 | 594 | 412 | 69（65.6-73.1） |
